# Comparison of Test–Retest Reliability of Sound Field Audiometry Between a Newly Designed System for Small Audiometric Booths and a Conventional Sound Field System

**DOI:** 10.3390/jcm15062351

**Published:** 2026-03-19

**Authors:** Hong Chan Kim, Hwan Min Kim, Young Mi Choi, Kyoung-Ho Park, Hyeon Sang Bark, Hyong-Ho Cho

**Affiliations:** 1 Department of Otolaryngology-Head and Neck Surgery, Chonnam National University Hospital, Gwangju 61469, Republic of Korea; 2 Department of Otolaryngology-Head and Neck Surgery, Chonnam National University Medical School, Gwangju 61469, Republic of Korea; 3 Department of Otolaryngology-Head and Neck Surgery, College of Medicine, Catholic University of Korea, Seoul 06591, Republic of Korea; 4 Division of Applied Photonics System Research, Advanced Photonics Research Institute (APRI), Gwangju Institute of Science and Technology (GIST), Gwangju 61005, Republic of Korea

**Keywords:** audiometry, hearing aids, hearing loss, speech perception, reproducibility of results

## Abstract

**Objective:** Sound field (SF) audiometry is widely used to evaluate aided hearing performance. This study compared the test–retest reliability and between-system equivalence of SF audiometry between a newly designed SF system for small audiometric booths and a conventional SF system. **Methods:** Thirty-nine adults using hearing aids (56 tested ears; 19 females [26 ears] and 20 males [30 ears]) underwent aided warble-tone audiometry (0.25, 0.5, 1, 2, and 4 kHz) and aided speech audiometry using a conventional SF system (two loudspeakers at 45° azimuth and 1 m distance) and a newly designed small-booth SF system (two height-adjustable loudspeakers at 45° azimuth and at 30 cm distance). The same sequence was repeated to assess test–retest performance. The test–retest differences were evaluated using paired *t*-tests, and the variability was summarized using the coefficient of variation (CV). Equivalence of aided warble-tone thresholds between systems was evaluated using two one-sided tests (TOST) with a ±10 dB margin. **Results:** Within each system, aided warble-tone thresholds and aided speech reception thresholds (SRTs) did not differ significantly between the test and the retest (all *p* > 0.05). The warble-tone thresholds were equivalent between systems within the predefined ±10 dB margin by TOST. Aided word recognition scores (WRSs) at 65 dB HL were higher in the newly designed system than in the conventional system (*p* < 0.0001). The CVs were low in both systems and were slightly lower in the newly designed system. **Conclusions:** When the listener-to-loudspeaker position is strictly controlled, SF audiometry provides stable test–retest results in hearing-aid users. The newly designed SF system for small audiometric booths produced aided thresholds equivalent to those of the conventional SF system and yielded higher WRSs at 65 dB HL under the tested conditions.

## 1. Introduction

Hearing loss is common among older adults and is associated with substantial functional limitations in daily life. Beyond communication difficulty, age-related hearing loss has been recognized as a potentially modifiable risk factor for dementia and cognitive decline. The Lancet Commission emphasized untreated hearing loss as a key target for dementia prevention strategies, and recent updates further strengthened the evidence by linking hearing rehabilitation with improved brain health outcomes [1,2]. In addition, the ACHIEVE randomized controlled trial suggested that a comprehensive hearing intervention, including appropriately fitted hearing aids, may slow cognitive decline in older adults who are at an increased risk [3]. Hearing loss has also been associated with impaired postural control and an increased fall risk, and hearing rehabilitation may provide benefits extending beyond communication [4].

Given these broader health implications, timely and appropriate hearing rehabilitation is important, and an accurate assessment of hearing-aid benefit is essential. In clinical practice, hearing-aid performance can be assessed using real-ear measurements (REMs) and aided sound field (SF) audiometry, along with patient-reported outcome measures [5].

The SF test is a method for evaluating hearing by delivering a sound signal generated by an audiometer to a subject through one or more speakers, rather than headphones, in an audiometric booth [6]. Since the stimulus sound is given through a speaker, it is more useful for infants and patients with developmental disabilities, for whom a general hearing test is difficult. It is also more useful than REM for evaluating the gain from cochlear implantation or bone conduction hearing aids [7]. However, SF testing has several practical limitations. A large soundproof booth (typically ≥2 × 2 m) may be required to meet the International Organization for Standardization (ISO) recommendations, and SF test–retest variability can be larger than that of REM, resulting in a wider reliability interval (SF test 15 dB vs. REM 3 dB) [8,9]. Recently, a newly designed SF audiometry system (SF-1^®^, Hankyul Healthcare, Gwangju, Republic of Korea) intended for small audiometric booths (approximately 1 × 1 m) was developed and shown to have a comparable performance to the conventional SF system [10].

Therefore, this study aimed to compare the test–retest reliability of aided SF audiometry between a conventional SF system and a newly designed SF system for small audiometric booths in hearing-aid users. We additionally evaluated whether the aided warble-tone thresholds obtained in the two systems were equivalent within a clinically acceptable margin using an equivalence testing framework.

## 2. Materials and Methods

### 2.1. Subjects

We enrolled 39 adults who used hearing aids and who visited two tertiary referral hospitals in Korea. In total, 56 ears were tested. The inclusion criteria were hearing-aid use and a maximum phonetically balanced word recognition score (PBmax) of >30%. The cohort included 39 adults (19 females, 26 tested ears; 20 males, 30 tested ears), with a mean age of 67.7 ± 9.8 years. Seventeen participants were bilateral and 22 were unilateral hearing-aid users; the testing was performed with the hearing aids worn in each participant’s usual configuration. Before hearing-aid use, the mean pure-tone audiometric threshold was 55.5 ± 10.5 dB HL. The mean SRT was 60.5 ± 11.8 dB HL, and the mean WRS was 71.5 ± 18.6% (Table 1).

Written informed consent was obtained from all participants. The study was approved by the Institutional Review Board of Chonnam National University Hospital (CNUH-2019-201).

### 2.2. Methods

**1**. **Sound field systems and calibration**

**Figure 1:**
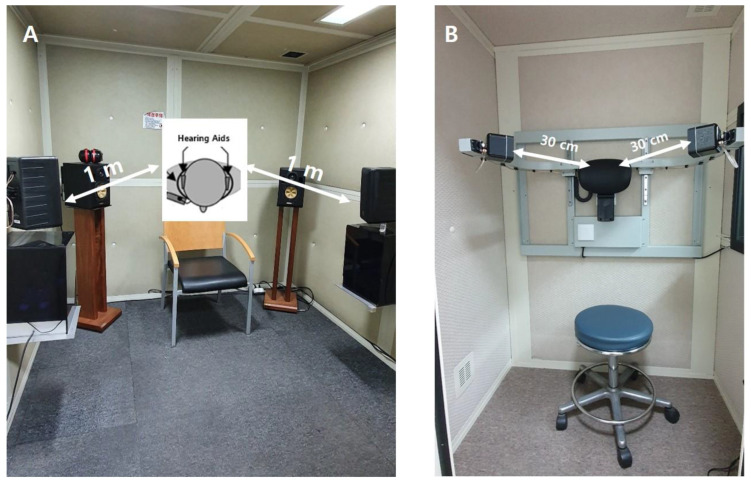
Sound field audiometry set-ups. (**A**) The conventional SF system in a 2 × 2 m audiometric booth with two fixed loudspeakers positioned 1 m from the participant at 45° azimuth. (**B**) The newly designed SF system in a 1 × 1 m booth with two height-adjustable loudspeakers positioned 30 cm from the participant’s ear level at 45° azimuth.

Two SF test configurations were evaluated (Figure 1):

The conventional SF system: The conventional loudspeaker arrangement (±45° azimuth at 1 m) was selected because it reflects a commonly used clinical sound field geometry and the recommended procedures for aided sound field assessment, providing symmetric presentation while minimizing head–shadow effects and excessive near-field variability. The warble tones and speech stimuli were delivered through the loudspeakers via an audiometer [6].

The newly designed SF system for small audiometric booths (SF-1^®^, Hankyul Healthcare, Gwangju, Republic of Korea): In the newly designed system, the height of two loudspeakers was adjusted to align with ear level, while the 30 cm distance and 45° azimuth were kept constant using fixed speaker mounts and a headrest. The loudspeaker height could be adjusted to match the participant’s ear level. The testing was performed in a 1 × 1 m audiometric booth.

All of the audiometric devices were calibrated according to standard procedures prior to the initiation of the study and were verified using routine (daily) checks. An additional verification was performed if any deviation was suspected.

**2**. **Test procedure**

**Figure 2:**
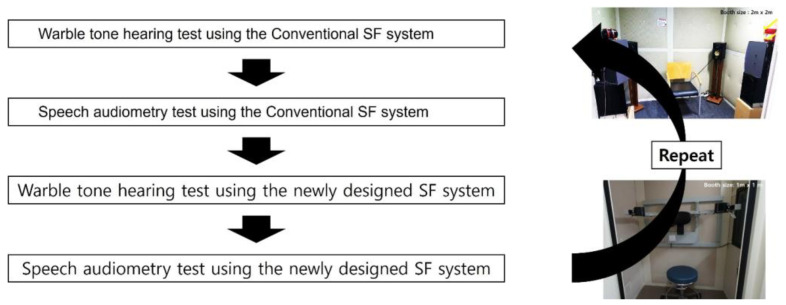
Test sequence. The participants were tested in the conventional SF system first, followed by the newly designed SF system; the same sequence was then repeated for test–retest assessment.

After confirming appropriate hearing-aid function, testing was performed in a standardized order (Figure 2). To minimize the variability from the participant’s head position relative to the sound source, participants were instructed to remain in a constant seated position for the conventional SF system and to place their head on a headrest for the newly designed system.

The test sequence was:

(1) Aided SF warble-tone thresholds at 0.25, 0.5, 1, 2, and 4 kHz in the conventional SF system.
(2) Aided SRT and aided WRS in the conventional SF system.
(3) Aided SF warble-tone thresholds at 0.25, 0.5, 1, 2, and 4 kHz in the newly designed SF system.
(4) Aided SRT and aided WRS in the newly designed SF system.
(5) Steps 1–4 were repeated to obtain test–retest measurements.

The tests and retests were conducted during the same session. The retest started immediately after completing the initial battery in both systems, resulting in an interval of approximately 20 min between test and retest for each outcome.

**3**. **Outcomes**

The warble-tone thresholds (dB HL) were measured at 0.25, 0.5, 1, 2, and 4 kHz. For comparison with common clinical reporting, a weighted four-frequency average was calculated as [500Hz + 2 × 1000Hz + 2 × 2000Hz + 4000Hz]/6. The speech audiometry outcomes included aided SRT (dB) and aided WRS (%) at 65 dB HL.

**4**. **Statistical Analysis**

A paired *t*-test was used to compare the differences in the test and retest audiometric results from the conventional and newly designed SF systems, with a *p*-value of <0.05 indicating a significant difference. Test–retest reliability was compared using coefficients of variation (CV%; [standard deviation/mean] × 100). Also, the equivalence of both systems was analyzed using two one-sided tests (TOST) with a margin of 10 dB (participants with hearing aids) to determine the statistical significance at the 95% confidence interval. The ±10 dB bound was selected as a pragmatic clinical tolerance for aided sound field threshold comparisons; however, the equivalence bounds may vary by clinical context. Because outcomes were measured repeatedly in the same participants/ears under two conditions (conventional vs. newly designed) and across two runs (test vs. retest) within the same session, paired statistical procedures were used to evaluate the systematic differences between the repeated measurements. Paired *t*-tests were applied to test whether the mean test–retest difference within each system deviated from zero. The variability was additionally summarized using the coefficient of variation (CV) to provide a scale-normalized measure of dispersion across frequencies. Between-system equivalence of aided warble-tone thresholds was evaluated using two one-sided tests (TOST) consistent with a clinically pragmatic tolerance for aided sound field threshold comparisons. All statistical analyses were performed using R software (version 4.0, R Foundation for Statistical Computing, Vienna, Austria).

**5**. **Use of Artificial Intelligence**

During the preparation of this manuscript, the authors used ChatGPT (OpenAI, GPT-5.2) solely for English language editing (e.g., grammar and wording). The AI tool was not used for study design, data analysis, data interpretation, or the generation of scientific content. All authors reviewed and approved the final manuscript and take full responsibility for its content.

## 3. Results

### 3.1. Test–Retest Aided Warble-Tone Thresholds in the Conventional SF System

**Figure 3:**
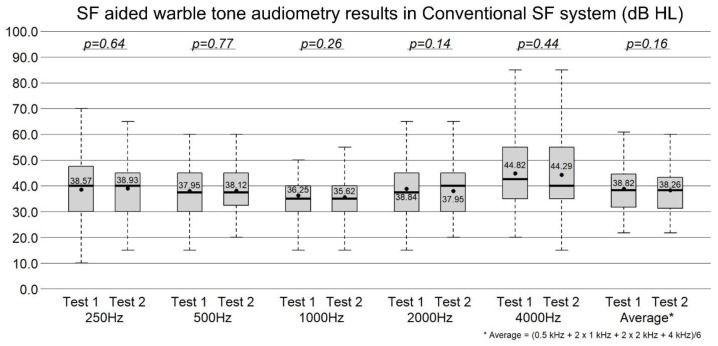
The test–retest aided warble-tone thresholds in the conventional SF system. No significant differences were observed between test and retest at any frequency (paired *t*-test).

In the conventional SF system, the mean aided warble-tone thresholds (test vs. retest) were 38.57 dB vs. 38.93 dB at 0.25 kHz (*p* = 0.64), 37.95 dB vs. 38.12 dB at 0.5 kHz (*p* = 0.77), 36.25 dB vs. 35.62 dB at 1 kHz (*p* = 0.26), 38.84 dB vs. 37.95 dB at 2 kHz (*p* = 0.14), and 44.82 dB vs. 44.29 dB at 4 kHz (*p* = 0.44). The weighted four-frequency average was 38.82 dB vs. 38.26 dB (*p* = 0.16) (Figure 3). No significant test–retest differences were observed at any frequency.

### 3.2. Test–Retest Aided Warble-Tone Thresholds in the Newly Designed Small-Booth SF System

**Figure 4:**
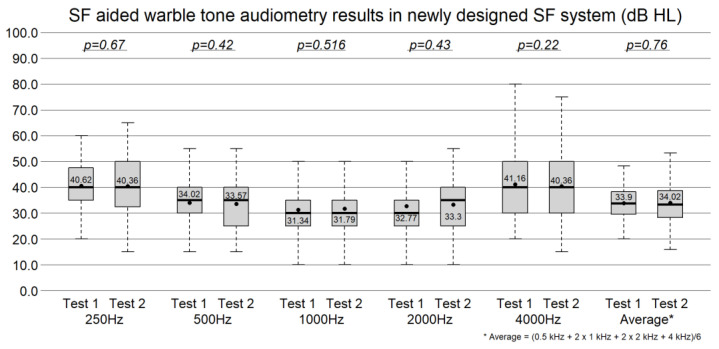
The test–retest aided warble-tone thresholds in the newly designed small-booth SF system. No significant differences were observed between test and retest at any frequency (paired *t*-test).

In the newly designed SF system, the mean aided warble-tone thresholds (test vs. retest) were 40.62 dB vs. 40.36 dB at 0.25 kHz (*p* = 0.67), 34.02 dB vs. 33.57 dB at 0.5 kHz (*p* = 0.42), 31.34 dB vs. 31.79 dB at 1 kHz (*p* = 0.52), 32.77 dB vs. 33.30 dB at 2 kHz (*p* = 0.43), and 41.16 dB vs. 40.36 dB at 4 kHz (*p* = 0.22). The weighted four-frequency average was 33.90 dB vs. 34.02 dB (*p* = 0.76) (Figure 4). No significant test–retest differences were observed at any frequency.

### 3.3. Test–Retest Aided Speech Audiometry in Both Systems

**Figure 5:**
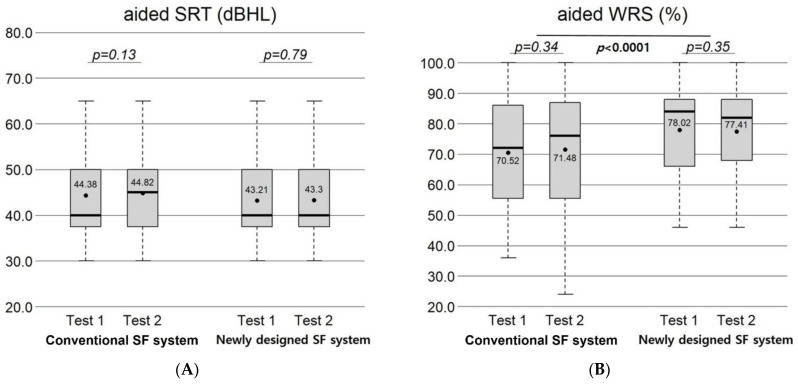
Aided speech audiometry in the conventional and newly designed SF systems. (**A**) The test–retest aided SRT did not differ within either system. (**B**) The test–retest aided WRS at 65 dB HL did not differ within either system; however, WRS at 65 dB HL was higher in the newly designed system than in the conventional system. (paired *t*-test).

For aided speech audiometry, the test vs. retest aided SRTs were 44.38 dB vs. 44.82 dB in the conventional SF system (*p* = 0.13) and 43.21 dB vs. 43.30 dB in the newly designed system (*p* = 0.79). The test vs. retest aided WRSs at 65 dB HL were 70.52% vs. 71.48% in the conventional SF system (*p* = 0.34) and 78.02% vs. 77.41% in the newly designed system (*p* = 0.35). When comparing systems, aided WRS at 65 dB HL was higher in the newly designed system than in the conventional system (*p* < 0.0001) (Figure 5).

### 3.4. Test–Retest Variability (Coefficient of Variation)

**Figure 6:**
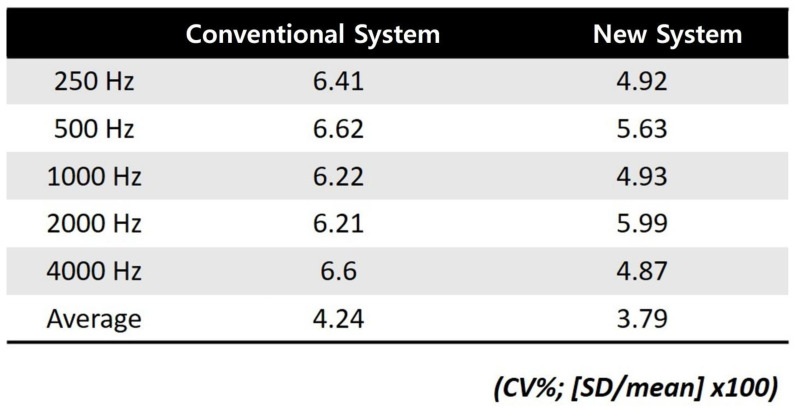
The coefficient of variation (CV%) for aided warble-tone thresholds in both systems. “Average” refers to the pure-tone average at four frequencies (PTA4). The results demonstrate a low test–retest variability.

The CVs for aided warble-tone thresholds in the conventional vs. newly designed system were 6.41% vs. 4.92% at 0.25 kHz, 6.62% vs. 5.63% at 0.5 kHz, 6.22% vs. 4.93% at 1 kHz, 6.21% vs. 5.99% at 2 kHz, and 6.60% vs. 4.87% at 4 kHz. For the weighted four-frequency average, the CVs were 4.24% vs. 3.79% (Figure 6).

### 3.5. Equivalence Testing Between Systems (TOST)

**Figure 7:**
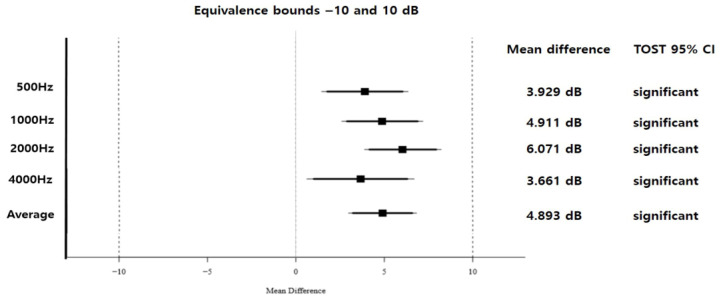
The equivalence testing (TOST) of aided warble-tone thresholds between systems. The results show equivalence within the predefined ±10 dB margin at the 95% confidence interval.

The mean differences in the aided warble-tone thresholds between the conventional and newly designed systems were 3.92 dB at 0.5 kHz, 4.91 dB at 1 kHz, 6.07 dB at 2 kHz, and 3.66 dB at 4 kHz, with a mean difference of 4.89 dB for the weighted four-frequency average. The TOST analyses demonstrated an equivalence between systems within the predefined ±10 dB margin at the 95% confidence interval (Figure 7).

## 4. Discussion

In this study, we compared the test–retest reliability of aided SF audiometry between a conventional SF set-up and a newly designed SF system intended for small audiometric booths. Across both systems, aided warble-tone thresholds and aided SRT showed no significant differences between the test and retest. In addition, the CVs were low across the tested frequencies, supporting stable repeated measurements when the participant’s position relative to the loudspeaker is controlled.

REM, SF systems, and questionnaires can be used to evaluate the functional gain from personal sound amplification products (PSAPs) or hearing aids [11,12]. Among them, SF audiometry is a relatively easy test that can be performed in a similar way to pure tone audiometry tests. It can be applied to infants, young children, or patients with disabilities who have difficulty with conventional hearing tests. It is also useful for evaluating the benefit of hearing aids in adults and children [7]. However, a key practical limitation of SF audiometry is that a large soundproof booth is often required to meet ISO recommendations [13]. Moreover, SF tests have been reported to have wider reproducibility intervals than REM, which can limit their utility for detecting small changes in aided performance [8,9,14,15]. The newly designed SF system addressed space constraints by enabling SF testing in a 1 × 1 m booth. In the current study, between-system equivalence testing using TOST with a ±10 dB margin demonstrated that aided warble-tone thresholds obtained with the newly designed system were equivalent to those obtained with the conventional SF set-up across the primary clinical frequencies (0.25–4 kHz) and for the weighted four-frequency average. These findings are consistent with previous work showing a comparable performance between a compact SF system and a conventional system [10].

Another concern is the reproducibility of the test. Thus, in the current study, we wanted to evaluate the reproducibility by checking the test–retest reliability using the two different SF test systems. The differences between the first and second tests in the test–retest reliability in both SF systems were not significant, indicating that the test–retest error was small (*p* > 0.05). In addition, when the reliability between the tests was compared using the coefficient of variation method, both types of testing equipment showed low variation, indicating test–retest reliability. Moreover, the SF system, newly designed for a smaller soundproof booth, showed a lower variation, and the reliability between the test and retest was superior compared to the conventional system. It showed a much smaller variation than that of the conventional SF system, in which a 15 dB interval was required for 95% confidence [15]. This might have resulted from placing the subject in the same position (in the case of the conventional system) or positioning the subject’s head in one position (in the case of the newly designed system). The test–retest reliability of the speech test using the SF system showed no significant differences in either system, confirming that the test was reliable. The SRT was similar between the two systems.

From a practical standpoint, our results indicate that when the source–listener geometry and head position are tightly controlled, aided sound field measures can remain stable within a session even in a space-limited booth. The equivalence of aided warble-tone thresholds suggests that the compact system can reproduce conventional aided threshold outcomes under standardized conditions, supporting its feasibility for clinics with limited audiometric space.

An additional observation was that the aided WRS at 65 dB HL was higher in the newly designed SF system than in the conventional system. The speech discrimination performance in SF testing may be influenced by room acoustics, including reverberation, and by the geometric relationship between the loudspeaker and the listener [6]. Because the conventional system used a 1 m distance and the newly designed system used a 30 cm distance, differences in the effective sound level at the listener position or in reverberant characteristics may have contributed to the observed difference. The higher aided WRS in the newly designed system may be related to tighter control on the head position and/or the differences in the acoustic environment that are associated with the shorter listener-to-loudspeaker distance. Therefore, the higher WRS in the newly designed system should be interpreted as a system-specific performance difference under the tested conditions rather than a definitive superiority in all clinical environments.

This study has several limitations. First, the system order was not randomized or counterbalanced; therefore, learning or fatigue effects may have influenced the speech outcomes. Second, some participants contributed data from both ears, which may introduce a within-subject correlation and limit the strict statistical independence at the ear level. Third, the reliability was assessed within the same session; additional metrics (e.g., ICC, Bland–Altman limits of agreement, and SEM/MDC with confidence intervals) and between-session retesting were not performed and should be considered in future studies. Finally, a more detailed and objective acoustic characterization (e.g., background noise levels and room acoustic metrics) would be valuable to better isolate distance- and room-related effects, particularly for speech recognition testing.

In summary, SF audiometry can provide a reproducible aided threshold and speech measures when the listener positioning is carefully controlled. The newly designed small-booth SF system produced aided thresholds equivalent to those obtained with the conventional SF system and may be useful in clinics with space-limited audiometric facilities.

## 5. Conclusions

Test–retest reliability of SF audiometry for hearing-aid evaluation can be achieved when the distance between the listener and the loudspeaker is kept constant. The newly designed SF system for small audiometric booths performed equivalently to the conventional SF system in aided warble-tone thresholds and yielded higher WRS at 65 dB HL under the tested conditions.

## Figures and Tables

**Table 1:** The demographic and baseline audiometric characteristics of participants.

	Female	Male	Total
**Patients**	19	20	39
**Ears**	26	30	56
Age (years) *	67.3 ± 7.8	68.1 ± 11.3	67.7 ± 9.8
PTA (dBHL) *	54.5 ± 12.4	56.3 ± 8.4	55.5 ± 10.5
SRT (dBHL) *	62.5 ± 10.7	58.8 ± 12.4	60.5 ± 11.8
WRS (%) *	74.7 ± 18.7	68.7 ± 18.1	71.5 ± 18.6

* Average ± Standard deviation.

## Data Availability

The data that support the findings of this study are available from the corresponding author upon reasonable request.

## Abbreviations

The following abbreviations are used in this manuscript:

SF	Sound field
CV	Coefficient of variation
REM	Real-ear measurements
TOST	Two one-sided tests
